# Increasing reverse total shoulder liner constraint in clinically planned cases causes decreased simulated range of motion and LIMIT, a novel phenomenon

**DOI:** 10.1016/j.jsea.2026.100070

**Published:** 2026-08-10

**Authors:** Joseph W. DiCecco, Adam Maestas, Axel Clement, Hugo Giambini, Matthew T. Glazier, Anil K. Dutta, Robert U. Hartzler

**Affiliations:** a UT Health San Antonio, San Antonio, TX; b Université Paris-Saclay, Paris, France; c University of Texas San Antonio, San Antonio, TX; d Burkhart Research Institute for Orthopaedics, San Antonio, TX; e The San Antonio Orthopaedic Group, San Antonio, TX

**Keywords:** Reverse total shoulder arthroplasty (rTSA), Polyethylene liner constraint, Range of motion (ROM) simulation, Prosthetic impingement, Scapular notching, Implant design and positioning

## Abstract

**Background:**

Optimization of reverse total shoulder arthroplasty (rTSA) component design and positioning can improve outcomes. Polyethylene liner constraint (depth/radius) influences prosthetic stability and simulated shoulder range of motion (sROM). Increased constraint may reduce sROM due to earlier polyethylene-to-bone contact. Given that Minimal Clinically Important Differences in shoulder sROM can be small (external rotation = 3°, abduction = 7°, flexion = 12°), even subtle changes may meaningfully impact outcomes. The purpose of this study was to characterize simulated virtual passive sROM in a series of clinically planned rTSA cases while varying liner constraint.

**Methods:**

Clinically planned and performed rTSA cases by a single surgeon (R.U.H.) between August 2020 and September 2024 were analyzed using Blueprint software. For each case, polyethylene liner constraint was varied (45%, 55%, and 65%) while all other implant parameters were held constant. Cases indicated for diagnoses without significant osteophytes (massive rotator cuff tear, avascular necrosis, or rheumatoid arthritis), as well as rotator cuff tear arthropathy cases without osteophytes, were included. For each simulation, the mechanism responsible for impingement at each end-sROM was visually determined and recorded. The primary outcome was simulated passive sROM in 6 planes. Post hoc multiple comparisons were performed using the Tukey procedure.

**Results:**

Seventy-three cases were included in the final analysis. Polyethylene-to-bone impingement occurred most frequently during simulated internal rotation, adduction, and external rotation. Increasing liner constraint from 45% to 65% significantly reduced sROM in internal rotation, external rotation, adduction, and flexion (Δ = 25.0°, 23.3°, 13.8°, 12.2°, respectively). Smaller interval increases in constraint (45% to 55% and 55% to 65%) also resulted in significant reductions in sROM across multiple planes. In 50 cases, increasing constraint resulted in conversion of the impingement mechanism to polyethylene-to-bone contact. This transition was associated with reductions in sROM of up to 177°, with a mean reduction of 58.7°.

**Discussion:**

sROM was most frequently affected, and to a greater magnitude, in adduction, external rotation, and internal rotation. Increasing constraint resulted in a distinct mechanical phenomenon, which we termed “LIMIT (Liner-Induced Mechanical Impingement Transition).” LIMIT describes the conversion of impingement type to polyethylene-to-bone as constraint increases. This occurred in 50 cases and was associated with substantial reductions in sROM, particularly in abduction, flexion, and extension. Increased constraint may contribute to scapular notching through heightened impingement during external rotation, internal rotation, and adduction. Importantly, reductions in sROM exceeded the Minimal Clinically Important Difference in external rotation and flexion, suggesting clinical relevance.

With reverse total shoulder arthroplasty (rTSA) now the most commonly performed type of shoulder arthroplasty worldwide, optimization of all procedural variables remains a priority in shoulder research.^16^ Despite its overall clinical success, rTSA is associated with several complications, including infection, acromial or scapular fractures, baseplate loosening, mechanical impingement, scapular notching, and instability.^5^^,^^7^^,^^21^^,^^28^ Instability has been reported as the most common complication following rTSA, with an incidence of 5%.^6^ Reducing instability may therefore decrease post-operative morbidity. Established risk factors for instability include obesity, male sex, subscapularis deficiency, prior surgery, surgical approach, bone deficiency, and prior trauma.^8^^,^^9^

Increasing humeral liner constraint is a common intraoperative strategy to enhance prosthetic stability.^13^ Greater constraint has been shown to improve stability under superior, posterior, and anterior loading conditions.^10^ A prior biomechanical study showed humeral cup depth as second only to compressive force in affecting stability.^14^ Furthermore, increasing constraint significantly increases the force required for dislocation.^8^ However, greater constraint has also been associated with scapular notching, a complication specific to rTSA. Scapular notching results from chronic mechanical impingement between the humeral liner and the scapular neck and contributes to adverse sequelae such as baseplate loosening, progressive glenoid bone loss, and ultimately implant failure.^18^^,^^20^ In addition to stability, rTSA influences post-operative range of motion (ROM). The Minimal Clinically Important Difference (MCID) represents “the smallest difference in a clinical outcome measure that a patient would perceive as beneficial and meaningful.” Simovitch et al^25^ reported MCID values of 3°, 7°, and 12° for external rotation, abduction, and forward flexion, respectively.

Existing 3-dimensional (3D) modeling studies evaluating the effect of constraint on simulated ROM (sROM) are limited by small sample sizes and the absence of real patient datasets.^12^ The purpose of this study was to investigate the effect of varying polyethylene liner constraint on impingement-free ROM in 6 planes using a large cohort of patient-specific 3D virtual models generated for pre-operative planning. We hypothesized that increasing humeral liner constraint would significantly affect simulated ROM.

## Methods

### Patients

This study is a retrospective review of patients who underwent rTSA performed by a single board-certified orthopedic surgeon (R.U.H.). All cases were pre-operatively planned using Blueprint software (Stryker, Kalamazoo, MI) with glenoid lateralization (via baseplate and/or glenosphere) and the minimal humeral lateralization philosophy using the Perform system. The glenoid side was planned so that the baseplate was low and the glenosphere provided at least 1-2 mm of overhang inferiorly for all cases. All cases were planned with the Tornier Perform humeral stem at 135° inclination and subjective “native” version. The study period spanned August 2020 to September 2024, including the entire duration for this implant system prior to study initiation. Shoulders were prospectively categorized by disease type in Blueprint by the operating surgeon. Inclusion criteria were diagnoses of massive rotator cuff tear, cuff tear arthropathy, avascular necrosis, or rheumatoid arthritis.

### Data collection

The UT Health San Antonio Institutional Review Board (IRB) determined that this study met criteria for exemption from IRB review (IRB ID: STUDY00001862). Three-dimensional bony anatomy for each shoulder was accessed using the most recent Blueprint desktop application (Version 4.2.1) by a Blueprint engineer (A.C.). Simulated ROM from 0° abduction was calculated in 6 planes: adduction, abduction, external rotation, internal rotation, extension, and flexion. Simulations were performed using 45%, 55%, and 65% humeral liner constraint within the Blueprint software. The humeral resting position was determined using a proprietary formula native to the Blueprint software and was applied uniformly to all cases.

**Figure 1:**
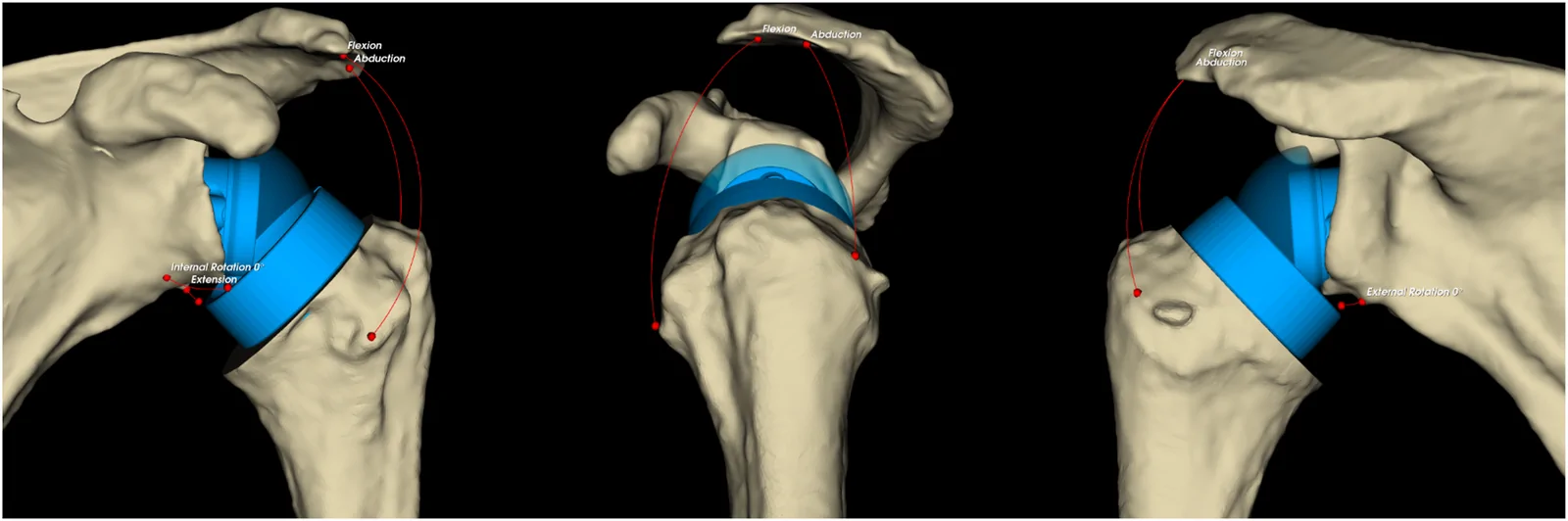
A visual from the Blueprint software with red arcs connecting 2 points of mechanical impingement.

For each constraint level, impingement type at simulated end-sROM was visually assessed and recorded across all 6 planes of motion (Fig. 1). Impingement type was categorized as bone-to-bone, polyethylene-to-bone, or “none,” based on the mechanism limiting motion. A designation of “none” indicated that simulated ROM exceeded the software's maximum sROM threshold. If impingement type changed with increasing constraint (eg, 45% to 55% or 55% to 65% constraint), this was recorded as a biomechanically simulated event. All other implant parameters were held constant in accordance with the operating surgeon's planned configuration for the actual case.

### Statistical analysis

sROM outcomes were obtained for 73 patients across 6 shoulder motions (abduction, adduction, extension, flexion, external rotation, and internal rotation) and 3 implant configurations (45%, 55%, and 65%). Statistical analyses were primarily performed using the complete dataset. To assess the robustness of the findings, 2 sensitivity analyses were conducted. First, outliers were identified independently for each motion-implant combination using Prism (GraphPad Software), and the analyses were repeated after excluding the identified outlying observations. Second, all observations from patients in whom any outlier was identified were excluded, and the analyses were repeated. Repeated measures ANOVA (Analysis of Variance) was implemented in the entire data set; mixed-effects models (Restricted Maximum Likelihood (REML)) with Geisser-Greenhouse's epsilon correction were used to account for repeated measurements across implant conditions and motion angles within subjects and to accommodate the missing observations introduced by the sensitivity analyses. Implant type and motion angle were treated as fixed effects. When appropriate, post hoc multiple comparisons were performed using Tukey's procedure with adjustment for multiple testing. redundant Differences within a specific motion and liner configuration are shown. Statistical significance was set at *P* < .05.

An outlier analysis identified 14 of 1,314 observations (1.1%), corresponding to 6 patients. Repeating the analyses after excluding the identified outlying observations, or after excluding all data from the 6 corresponding patients, did not alter the overall interpretation of the results. Therefore, the primary results presented are based on the complete dataset.

## Results

**Figure 2:**
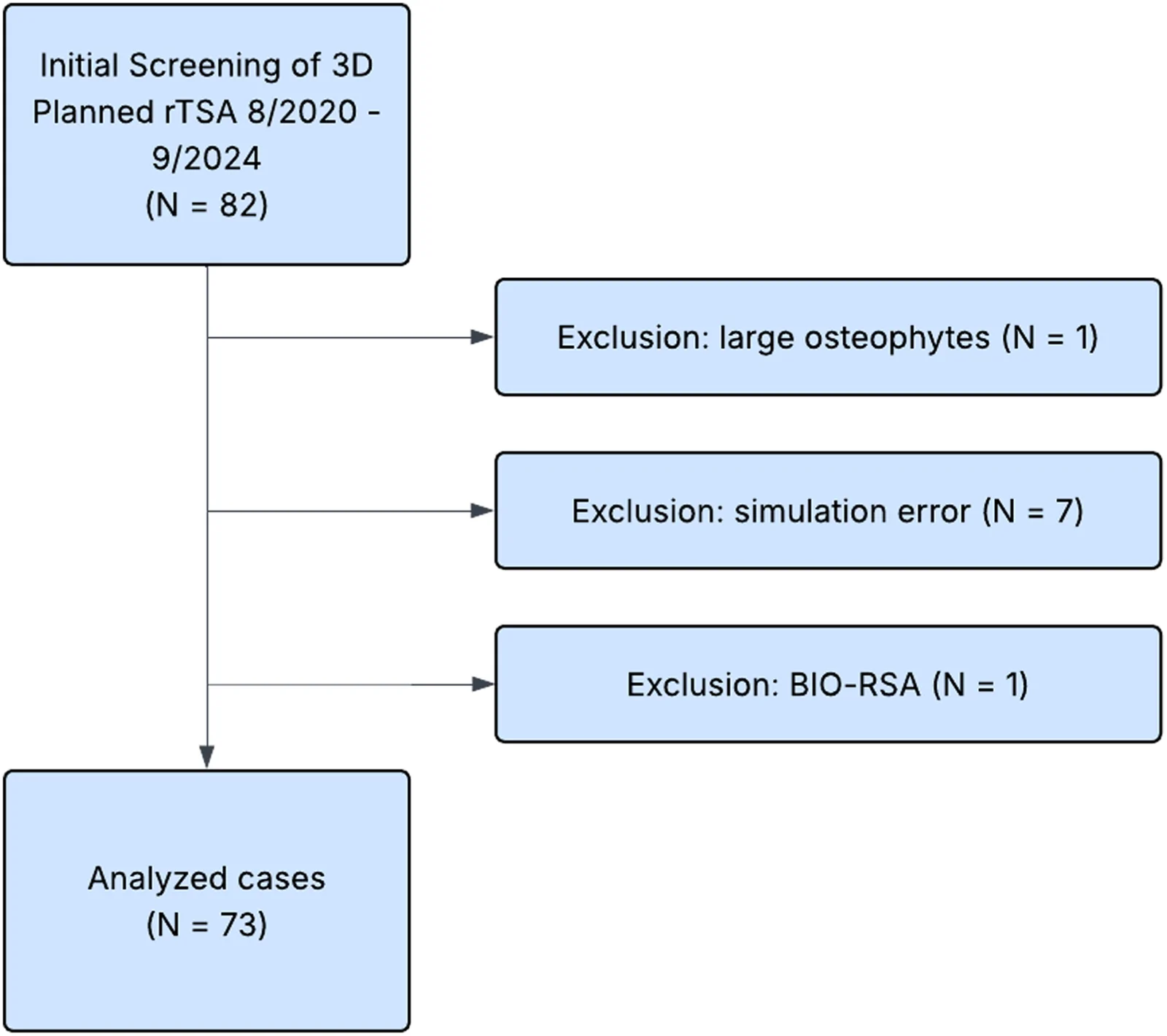
Exclusions. *3D*, 3-dimensional; *rTSA*, reverse total shoulder arthroplasty.

Eighty-two shoulders met initial inclusion criteria after a query of the planning software database. Two cases involving marginal osteophytes and bone graft reconstruction were visually identified and excluded (Fig. 2). An additional 7 shoulders were excluded due to incompatibility between older file types and new Blueprint software sROM simulation parameters. The final study included 73 shoulders.

Demographic and planned implant characteristics of the included shoulders are described in Table I. Diagnoses of included patients were massive rotator cuff tears (n = 33), rotator cuff arthropathy (n = 32), rheumatoid arthritis (n = 7), and avascular necrosis (n = 1). The mean glenoid implant version was −4.2 ± 4.2.

**Table I:** Sample descriptive statistics.

Descriptive Statistic	Value
Laterality, n (%)	
Right	49 (67)
Left	24 (33)
Sex, n (%)	
Male	32 (44)
Female	41 (56)
Age (yr), mean ± Standard Deviation (SD) (range)	71.0 ± 9.7 (33-86)
Height (cm), mean ± SD (range)	166.6 ± 9 (147-193)
Weight (kg), mean ± SD (range)	79.7 ± 19.0 (43-132)
Body Mass Index (BMI) (kg/m^2^), mean ± SD (range)	28.1 ± 5.9 (18.4-46.1)
Glenosphere size, n	
33 mm	12
36 mm	32
39 mm	25
42 mm	4
Glenosphere lateralization, n	
Non-lateralized	40
Lateralized	33
Glenoid baseplate	
Standard (+3 mm lateralization)	39
Full wedge (+6 mm lateralization)	34
Glenoid version (°) , mean ± SD (range)	−6.5 ± 8.4 (−28 to 30)
Glenoid inclination (°), mean ± SD (range)	9.5 ± 7.3 (−7 to 41)
Glenoid implant version (°), mean ± SD (range)	−4.2 ± 4.2 (−13 to 4)
Glenoid implant inclination (°), mean ± SD (range)	2.1 ± 4.1 (−10 to 17)

Bone-to-bone impingement was the most common impingement type in abduction, flexion, and extension, while polyethylene-to-bone impingement prevailed in adduction, external rotation, and internal rotation in all but 1 case (Table II). In both extension and flexion, 5% of cases in each category had no impingement. A change in impingement type with increasing constraint, which we termed the LIMIT (Liner-Induced Mechanical Impingement Transition) phenomenon, occurred in flexion, extension, and abduction at proportions of 5%, 4%, and 3%, respectively, for a total of 9 cases. When changing from 55% to 65% constraint, an additional 30%, 12%, and 14% of all shoulders underwent LIMIT-redundant. Impingement-type change usually started as bone-to-bone impingement but in 10 total cases converted from no impingement (“none”). Impingement type always converted to polyethylene-to-bone upon transitioning to the higher constraint ratio (Table II).

**Table II:** Number of impingement types in each plane of motion.

Plane of Motion	Bone-to-bone, n (%)	Polyethylene-to-bone, n (%)	None, n (%)	45%-55% change, n (%)	55%-65% change, n (%)
Abduction	59 (81)	2 (3)	0 (0)	2 (3)	10 (14)
Adduction	0 (0)	73 (100)	0 (0)	0 (0)	0 (0)
Flexion	38 (52)	5 (7)	4 (5)	4 (5)	22 (30)
Extension	56 (77)	1 (1)	4 (5)	3 (4)	9 (12)
External rotation	1 (1)	72 (99)	0 (0)	0 (0)	0 (0)
Internal rotation	0 (0)	73 (100)	0 (0)	0 (0)	0 (0)

**Figure 3:**
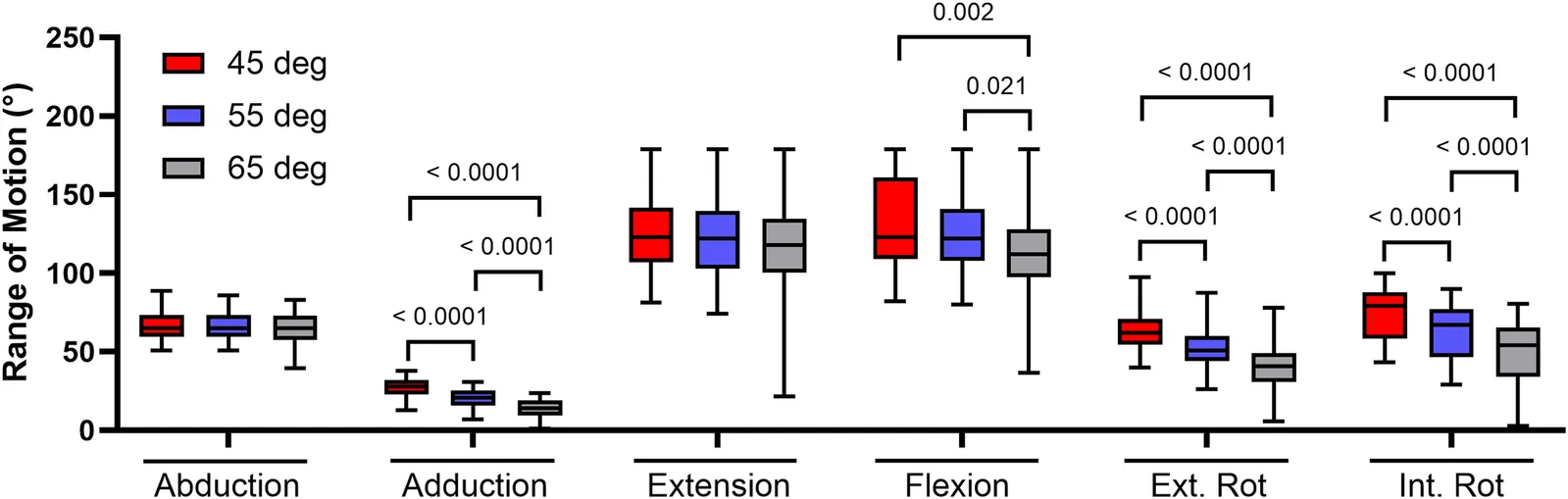
Effect of increased constraint on simulated ROM. *ROM*, range of motion.

Increasing humeral liner constraint yielded significant decreases in sROM in 4 out of 6 planes (Table III, Fig. 3). Increasing liner constraint from 45% to 55% had the most pronounced effect on internal rotation and external rotation, with significant decreases in sROM of 11.5° and 11.4°, respectively. Adduction also demonstrated a significant reduction in sROM of 7.1°. This pattern held when increasing constraint from 55% to 65%, with differences of 13.5° and 12.0° for internal and external rotation, respectively. Flexion and adduction also demonstrated less pronounced but significant decreases in sROM in this interval. On the other hand, abduction and extension did not show significant differences with increased constraint in any interval.

**Table III:** Changes in simulated ROM across planes of motion.

Plane of Motion	Mean (°)			Delta (°)			*P* value		
	45% constraint	55% constraint	65% constraint	45%-55%	55%-65%	45%-65%	45%-55%	55%-65%	45%-65%
Abduction	66.7	66.4	65.5	0.3	0.8	1.1	.899	.080	.188
Adduction	27.5	20.4	13.7	**7.1**	**6.7**	**13.8**	**<.0001**	**<.0001**	**<.0001**
Extension	126.1	123.1	119.3	3.0	3.1	6.3	.982	.443	.628
Flexion	129.9	126.3	117.4	3.6	**8.4**	**12.2**	.322	**<.001**	**<.001**
External rotation	64.3	53.0	41.0	**11.4**	**12.0**	**23.3**	**<.0001**	**<.0001**	**<.0001**
Internal rotation	74.5	63.0	49.5	**11.5**	**13.5**	**25.0**	**<.0001**	**<.0001**	**<.0001**

*ROM*, range of motion.

Statistically significant (*P* < 0.05) deltas and their corresponding *P* values are bolded.

Impingement type changed in 9 cases when increasing constraint from 45% to 55%. The mean change in sROM across all planes of motion for these cases was 42.8°, with a range of 2° - 130°. In the 41 cases where impingement type changed from 55% to 65%, the mean change in sROM across all planes of motion was 38.8°, with a range of 1° - 177°. Among these 41 cases, change occurred most frequently in flexion (n = 22, mean change = 39.2°), whereas the largest mean change in sROM was observed in extension (n = 9, mean = 62.2°).

## Discussion

In this large clinical series of 3D-planned rTSA, the effects of polyethylene liner constraint on simulated ROM and impingement type were investigated. Increasing liner constraint reduced sROM most frequently and most drastically in adduction, external rotation, and internal rotation. This study is unique in that impingement mechanisms were categorized and analyzed.

During the course of the study, an unexpected phenomenon (LIMIT) was observed: in some cases, impingement type changed as constraint increased. This has not been previously described in published literature. LIMIT only occurred in those planes of motion constrained by bone-to-bone contact (abduction, flexion, and extension). LIMIT was rare when increasing the constraint from 45% to 55%. Even so, LIMIT frequently resulted in large decreases in sROM, with a mean reduction of 42.8°, and a maximum of 130°. LIMIT was much more common when constraint increased from 55% to 65%. However, the average reduction in sROM (mean = 38.8°) was less pronounced than in the prior interval, although still far greater than established MCID thresholds. Reductions in simulated ROM of this magnitude are likely to translate clinically because they would produce mechanical impingement before soft tissue restraint. As such, pre-operative and intraoperative decision-making should weigh the potential for drastic reductions in sROM due to LIMIT against the benefits of increased constraint. LIMIT could lead to drastic, unexpected reductions in sROM. It is not clear why LIMIT was more common in flexion (n = 22) or more drastic in extension (mean = 62.2°). Future studies could investigate a potential correlation between scapular anatomy and LIMIT.

The MCID for external rotation is 3°. Each 10% increase in constraint (45% to 55% and 55% to 65%) resulted in an 11° and 12° reduction in simulated external rotation, totaling 23° of sROM reduction for a 20% increase in constraint (45% to 65%). While an MCID of 3° is relatively small and may not be necessarily noticed by the patient, each interval increase in constraint produced sROM reductions that far exceeded the MCID threshold for external rotation. These changes may be noticed by the patient.^25^ The MCID for flexion is 12°. Only a 20% increase in constraint resulted in an sROM reduction that exceeded this threshold, at 12.2° of reduction in flexion sROM.

The current study found a significant reduction of sROM in external rotation, internal rotation, adduction, and flexion, the planes most constrained by polyethylene-to-bone impingement. Some studies have had similar results: Nakazawa et al^19^ also found reduction in sROM for extension, in addition to adduction, external rotation, and internal rotation using a constrained liner in their computed tomography scan-based virtual ROM simulation in different software. Abdulla et al,^1^ a biomechanical study using fresh-frozen cadaveric shoulders, found reduction in external rotation only. They discussed that terminal impingement may be tuberosity-related rather than polyethylene. Our study helps confirm this, with 69 out of 73 cases impinging bone-on-bone in abduction.^1^ However, in a similar computer simulation, Gutiérrez et al^15^ found reduction in abduction with increased constraint, especially with inferior positioning of the glenosphere on the glenoid, with a milder effect on abduction ROM with superior positioning. The discrepancy between our results could be explained by general shifts in trend in rTSA planning in the last decade, such as increased lateralization and inferior tilt. Although inferior glenosphere positioning has also increased in recent times, perhaps increasing lateralization and inferior tilt have outweighed this effect in our study.^3^^,^^27^ Patel et al^22^ found decreased external and internal ROM associated with increased combined retroversion of the humeral and glenoid prostheses. Our study did not evaluate for any such effect, although mean glenoid component retroversion was modest at 4.2° (Table I). Taken together, these findings suggest that the relationship between constraint and ROM is complex and likely influenced by implant design, scapular anatomy, and evolving surgical techniques. Future studies investigating this effect are warranted.

Scapular notching is defined as glenoid neck erosion caused by repetitive mechanical abutment of the humeral component with the scapular neck. Scapular notching has been shown to have a negative impact on implant survivorship.^11^^,^^26^ Scapular notching is the most common complication of rTSA, occurring in 44-96% of cases.^17^^,^^20^ As such, this study helps determine and quantify the effect of constraint on scapular notching. The idea of scapular notching being a product of external rotation and internal rotation, in addition to adduction, has been discussed since 2011.^17^^,^^20^ Our results confirm this, with the humeral liner making contact with the scapula in 99% of cases in external rotation and in 100% of cases in internal rotation. A 2012 radiographic study by Kowalsky et al^17^ found no significantly increased risk in scapular notching with increased constraint. Our findings are contradictory, with polyethylene-to-bone impingement dominating in adduction, external rotation, and internal rotation. The reason for our contradictory findings is unknown but may be due to using a different implant system, due to the aforementioned shifts in rTSA, or due to a discrepancy between computer-determined polyethylene impingement on the scapular neck and radiographically noticeable bone loss of the scapular neck. Perhaps polyethylene impingement has variable effects on scapular notching. Future biomechanical studies could help further determine and confirm the role of increased constraint in scapular notching.

Post-operative ROM can be difficult to predict, with intraoperative forward flexion being the most reliable predictor.^24^ This study may not be useful as a predictor of post-operative ROM, except in cases of large reductions in sROM, such as those involving LIMIT, in which a large reduction in ROM may cause bony impingement before soft tissue begins to restrain movement. In thinner patients, bony impingement may also be the predominate impingement mechanism in planes such as adduction, where soft tissue restriction is minimal. In a clinical study, Goodloe et al^13^ found no significant differences in post-operative ROM with increased constraint. This is likely due to the fact that soft tissue restricts glenohumeral motion before bony impingement can occur. However, Goodloe et al did not study the effects of increased constraint on adduction ROM. Future studies should evaluate the effect of increased constraint on clinical adduction ROM, since this plane of motion is minimally restrained by soft tissue involvement and presumably experiences bony impingement frequently, as evidenced by the scapular notching phenomenon.

This study has some limitations. First, this study was limited by lack of soft tissue involvement. Furthermore, simulated motion may differ from actual glenohumeral motion and does not factor in scapulothoracic motion, which is an important contributor to overall shoulder mobility and is one reason our simulated results differ from clinical observations.^13^ Second, this study was limited by analysis with the humerus in the adducted position only. Future studies could study rotational impingement patterns with the humerus at 90° of abduction. Third, we did not control for anatomical differences between patients. Arashiro et al^2^ and Patel et al^23^ found that scapular neck length and inferior tilt of the glenoid, respectively, can affect adduction ROM. Fourth, the design of this study held all other implant parameters, such as glenosphere and baseplate positioning, constant in order to isolate the effect of liner constraint. In an actual case, the surgeon obviously has the freedom to adjust these parameters. However, this may reflect a common clinical situation in which all final implant components have been placed except for the liner. Finally, 78% of rTSA cases deviate from pre-operative plans intraoperatively.^4^ Highlighting this fact helps contextualize the limited utility of our findings.

This study may be most useful in intraoperative decision-making as a broad characterization of the planes which are most likely to be affected by increased constraint and the degree to which they are affected. This study also points out a new phenomenon, LIMIT, which may severely decrease ROM when impingement type changes. Future studies with larger data sets could isolate more of these cases to help explain its cause.

## Conclusion

This study aimed to determine whether increased humeral liner constraint affects impingement-free sROM in rTSA. The null hypothesis was that increasing constraint would not significantly alter ROM. Increasing liner constraint resulted in significant reductions in adduction, external rotation, internal rotation, and flexion. We identified a novel phenomenon, LIMIT, which can lead to large reductions in ROM. These findings suggest that while increased constraint enhances stability, its benefits need to be weighed against reductions in ROM in key functional planes and contribution to polyethylene-to-bone impingement patterns associated with scapular notching.

## Disclaimers:

Funding: No funding was disclosed by the authors.

Conflicts of interest: Robert Hartzler reports consulting fees from Stryker. Anil Dutta reports consulting fees from Zimmer Biomet and Enovis. Axel Clement reports being a full-time employee of Stryker. Any additional authors, their immediate families, and any research foundations with which they are affiliated have not received any financial payments or other benefits from any commercial entity related to the subject of this article.
